# STRATEGIES TO CREATE CAREGIVER-FRIENDLY WORKPLACES

**DOI:** 10.1093/geroni/igac059.1131

**Published:** 2022-12-20

**Authors:** Eileen Tell, Pamela Nadash, Siena Ruggeri

**Affiliations:** ET Consulting, LLC, Belmont, Massachusetts, United States; University of Massachusetts Boston, Boston, Massachusetts, United States; Community Catalyst, Boston, Massachusetts, United States

## Abstract

An estimated one in six employees is juggling work and being a family caregiver. Research suggests working caregivers neglect their own health and experience higher levels of stress and poor mental health. Their employers also experience negative outcomes, most typically in terms of increased absenteeism, lost productivity, difficulty recruiting and retaining workers, and higher health care claim costs. A wide range of strategies have emerged for employers to better support working caregivers and, hopefully, thereby reducing the financial impact both for the company and the caregiver. In this session, we discuss recommendations that emerged from research with both public and private sector stakeholders to identify best practice models and action steps for a national strategy supporting working family caregivers. Recommendations range from employer education, voluntary recognition programs, tax credits for employer-paid caregiver support programs, expanded PFMLA, and even federal LTSS financing reform.

